# Brain Region and Cell Compartment Dependent Regulation of Electron Transport System Components in Huntington’s Disease Model Mice

**DOI:** 10.3390/brainsci11101267

**Published:** 2021-09-24

**Authors:** Johannes Burtscher, Giuseppe Pepe, Federico Marracino, Luca Capocci, Susy Giova, Grégoire P. Millet, Alba Di Pardo, Vittorio Maglione

**Affiliations:** 1 Institute of Sport Sciences, University of Lausanne, CH-1015 Lausanne, Switzerland; gregoire.millet@unil.ch; 2 Department of Biomedical Sciences, University of Lausanne, CH-1015 Lausanne, Switzerland; 3 IRCCS Neuromed, 86077 Pozzilli, IS, Italy; g.pepe1604@gmail.com (G.P.); federicomarracino@gmail.com (F.M.); luca.capocci@virgilio.it (L.C.); susygiova95@gmail.com (S.G.); dipardoa@hotmail.com (A.D.P.)

**Keywords:** Huntington’s disease, mitochondria, oxidative phosphorylation, neurodegeneration, striatum, cortex

## Abstract

Huntington’s disease (HD) is a rare hereditary neurodegenerative disorder characterized by multiple metabolic dysfunctions including defects in mitochondrial homeostasis and functions. Although we have recently reported age-related changes in the respiratory capacities in different brain areas in HD mice, the precise mechanisms of how mitochondria become compromised in HD are still poorly understood. In this study, we investigated mRNA and protein levels of selected subunits of electron transport system (ETS) complexes and ATP-synthase in the cortex and striatum of symptomatic R6/2 mice. Our findings reveal a brain-region-specific differential expression of both nuclear and mitochondrial-encoded ETS components, indicating defects of transcription, translation and/or mitochondrial import of mitochondrial ETS components in R6/2 mouse brains.

## 1. Introduction

Despite their heterogeneity, neurodegenerative diseases share several common features, including selective vulnerability of specific neuronal circuits and mitochondrial dysfunction. In the case of Huntington’s disease (HD), striatal medium spiny neurons are most vulnerable, although other brain regions are also affected by neurodegeneration [1]. Progressive motor, cognitive and psychiatric symptoms characterize the autosomal dominant disorder HD, and it is further associated with deficits of numerous peripheral organs and tissues such as skeletal muscle [2,3], heart [4,5], gastrointestinal tract, liver and adipose tissue, as recently reviewed [6]. The disease-causing mutation is an expansion of the trinucleotide CAG repeat in the Huntington gene [7,8].

Like in other neurodegenerative diseases, mitochondrial damage, both structurally [9] and functionally [10], characterizes HD brain, rendering therapeutic strategies targeting mitochondria attractive approaches in HD [11]. Despite converging features of mitochondrial dysfunction [12] and other metabolic deficits in neurodegenerative diseases [13], mitochondria in HD seem to be affected differentially. More specifically, important examples are the central role of mitochondrial complex II damage in HD, impaired mitochondrial import and deranged calcium homeostasis. 

Inhibition of complex II by the HD-causing mutated Huntingtin (Htt) protein [14], as well as by HD-like pathology induced by pharmacological inhibition of complex II, e.g., by 3-nitropropionic acid (3-NP) [15], support the notion that deterioration of the complex II function is central in HD pathogenesis. Intriguingly, we previously reported an especially prominent reliance on complex II-mediated mitochondrial respiration in the striatum of adult wild-type mice, which we hypothesized may furnish an explanation for the specific vulnerability of striatum in HD [16].

Unlike the other protein complexes of the electron transport system (ETS), the main subunits of complex II are all encoded by nuclear DNA, rendering complex II particularly vulnerable to impaired mitochondrial import. Deficits in mitochondrial import are indeed another well-established feature of HD and have been reported to be based on the interaction of mutated Htt with proteins of the inner mitochondrial membranes such as TIM23 [17,18].

Defects in the calcium homeostasis are well established in HD and are directly related to mutated Htt [19]. The precise mechanisms and consequences are becoming increasingly better understood. For example, a recent study demonstrates a dysregulation of calcium homeostasis in HD-model stem cells, indicating impaired neurogenesis and neuronal differentiation [20]. 

Although both mitochondrial dysfunction and selective neuronal vulnerability are well-described features of HD brain, whether mitochondrial damage is a cause or consequence of the neurodegenerative process [21] and why specific neurons are more vulnerable than others, as well as which aspects distinguish HD mitochondrial dysfunction from deficits observed in other neurodegenerative diseases, remain open questions. The latter point also includes the potential loss-of-function effects by mutation or aggregation of Htt that have been suggested to support mitochondrial function in health [22,23]. To better understand these peculiarities, more knowledge of the etiology and progression of HD with consideration of outcomes in different brain regions is required. 

To this end, we recently explored the possibility that mitochondrial respiration in the HD brain might be altered during disease progression and that these alterations may differ from one brain region to another. We studied these questions in a commonly used rodent model of HD [24], in R6/2 mice (overexpressing human N-terminal Htt, with around 160 glutamine repeats). In line with previous results of no [25], very modest [26] or functionally insignificant [27] respirational deficits in these mice, we also observed no significantly impaired mitochondrial respiration in symptomatic R6/2 mice [28]. There were, however, brain-region-dependent developmental changes in mitochondrial respiration that were slightly different between wild-type and R6/2 mice. 

Based on these findings, we speculated that either in vitro and ex vivo approaches to determine mitochondrial respiration may not accurately reflect in vivo respiration in HD models [28,29] or that compensatory mechanisms are at work during the disease pathogenesis, which mask mitochondrial respiration abnormalities, but possibly contribute to disease development or progression. Such compensation may be effectuated, for example, by altered gene expression or protein levels of ETS components. 

To explore this possibility, in the present study we investigated mRNA and protein levels of selected subunits of ETS complexes and ATP-synthase in two HD-relevant brain regions, the cortex and the striatum in symptomatic R6/2 mice. We hypothesized that R6/2 mice compensate for underlying metabolic deficits with alterations in the abundance of mRNA and proteins of ETS in these HD pathology-prone brain regions. To get a clearer idea about potential mechanisms of impaired transcription and translation of both nuclear and mitochondrial proteins, as well as on mitochondrial import of nuclear proteins, we analyzed proteins of nuclear and mitochondrial DNA origin. 

## 2. Materials and Methods

### 2.1. Animals

R6/2 HD mice (carrying approximately 160 ± 5 CAG repeat expansion) were housed in the animal facility at IRCCS Neuromed. Animal studies were approved by the Institutional Review Board Committee and Istituto Superiore di Sanità (ISS permit number: 760/2020-PR) and performed according to EU Directive 2010/63/EU for animal experiments. All the analyses were performed on symptomatic (11-week-old) mice. Both male and female mice were used.

### 2.2. Quantitative Real-Time PCR (qPCR)

Total RNA was extracted as previously described [30]. qPCR analysis was performed on a CFX Connect Real-Time System instrument (Bio-Rad Laboratories, Hercules, CA, USA). The following primers were used (5′→3).

Ndufa2 Fw: GCACACATTTCCCCACACTG; Ndufa2 Rv: CCCAACCTGCCCATTCTGAT; Sdha Fw: GGAACACTCCAAAAACAGACCT; Sdha Rv: CCACCACTGGGTATTCAGTAGAA; Uqcrc Fw: ACGGTGGGAGTGTGGATTGAC; Uqcrc Rv: CATTGCCAGGCCGATTCTTTG; CoxIV Fw: CCAAGTGGGACTATGACAAGAA; CoxIV Rv: AGCATGGACCATTGGATACG; Atp5b Fw: AGTTGCTGAGGTCTTCACGG; Atp5b Rv: CTTTGCCACGGCTTCTTC; Mt-nd1 Fw: AGGGTACATACAACTACGAAAAGGCC; Mt-nd1 Rv: AGTATTTGGAGTTTGAGGCTCATTC; Cyt-b Fw: TTATCGCGGCCCTAGCAA; Cyt-b Rv: TTTAATCCTGTTGGGTTGTTTGATC; CoxI Fw: CCTTTGCTTCAAAACGAGAA; CoxI Rv: ATAGGTTGGTTCCTCGAATG; Atp8 Fw: AGTCTCATCACAAACATTCCC; Atp8 Rv: GTTAGTGATTTTGGTGAAGGTG; Cyc Fw: GAGCTGTTTGCAGACAAAGTTC; Cyc Rv: CCCTGGCAXATGAATCCTGG. mRNA expression was normalized over Cyclophilin (Cyc).

### 2.3. Brain Lysate Preparation and Immunoblottings

Mice were sacrificed by cervical dislocation and brains were removed from the skull, weighed and bisected. Brains were immediately snap-frozen in liquid N2 and pulverized in a mortar with a pestle as previously described [30]. 

Proteins (20 µg) were resolved on 10% SDS-PAGE and immunoblotted with the following antibodies: anti-MT-ND1 (1:1000) (Abcam, Cambridge, UK, Cat. N. AB74257), anti-COX IV (1:1000) (Cell Signaling, Danvers, MA, USA, Cat. N. 4850), anti-SDHA (1:1000) (Abcam, Cat. N. AB14715), anti-Cytochrome C (1:1000) (BD Biosciences, San Jose, CA, USA, Cat. N. 556433).

For protein normalization, anti-Actin (1:5000) (Sigma Aldrich, St. Louis, MO, USA, Cat. N. A5441) and anti-Cyclophilin (1:2000) (Immunological Sciences, Rome, Italy, Cat. N. AB-83838) were used. Protein bands were visualized by ECL Plus (GE Healthcare, Chicago, IL, USA) and quantitated with Image Lab Software (Bio-Rad Laboratories).

### 2.4. Statistics 

All data were expressed as mean ± SD. Two-tailed unpaired *t*-tests were used for comparisons.

## 3. Results

### 3.1. Nuclearly Encoded Mitochondrial ETS Genes Are Differentially Expressed in the Brain of R6/2 Mice

We recently reported age-related changes in respirational capacities of different brain regions in the R6/2 HD mouse model [28].

In order to potentially correlate such changes with a differential expression of mitochondrial genes, we investigated the expression of selected subunits of ETS components and ATP-synthase (see Table 1) in both the cortex and striatum of symptomatic (11-week-old) R6/2 mice and wild-type (WT) littermates. 

**Figure 1:**
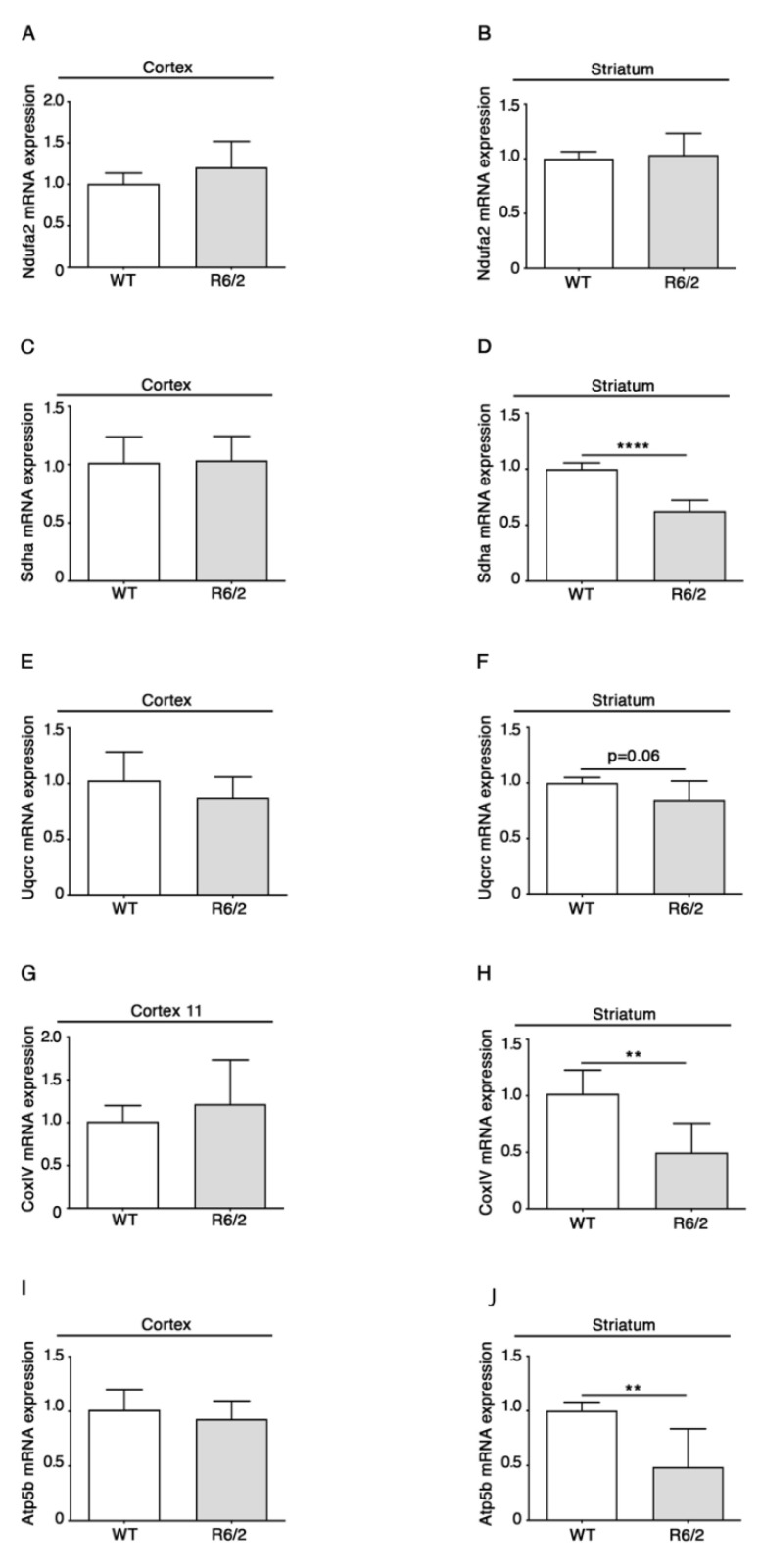
Altered RNA levels of components of the nuclear-encoded ETS and ATP-synthase in brains of 11-week-old R6/2 mice. qPCR analysis of both cortical and striatal Ndufa2 (**A**,**B**), Sdha (**C**,**D**), Uqcrc (**E**,**F**), Cox IV (**G**,**H**), Atp5b (**I**,**J**) are presented. N = 5/6 for each group of mice. Data are represented as mean ± SD. ** *p* < 0.01, **** *p* < 0.0001 (Unpaired *t*-test). The raw data used for generating the figure are reported in the Supplementary Materials Table S1.

qPCR analyses revealed no changes in Ndufa2 (complex I) (Figure 1A,B) and Uqcrc (complex III) (Figure 1E,F) RNAs for either the R6/2 cortex or striatum as compared to the control littermates.

Conversely, RNA levels of Sdha (complex II) (Figure 1C,D), Cox IV (complex IV) (Figure 1G,H) and Atp5b (complex V) (Figure 1I,J) were reduced in the R6/2 striatum but not the cortex (Figure 1C,D). 

### 3.2. Mitochondrially Encoded ETS Genes Are Also Differentially Expressed in the Brain of R6/2 Mice

Next, we investigated the expression levels of mitochondrially encoded genes in both the cortex and striatum of HD mice and WT littermates.

**Figure 2:**
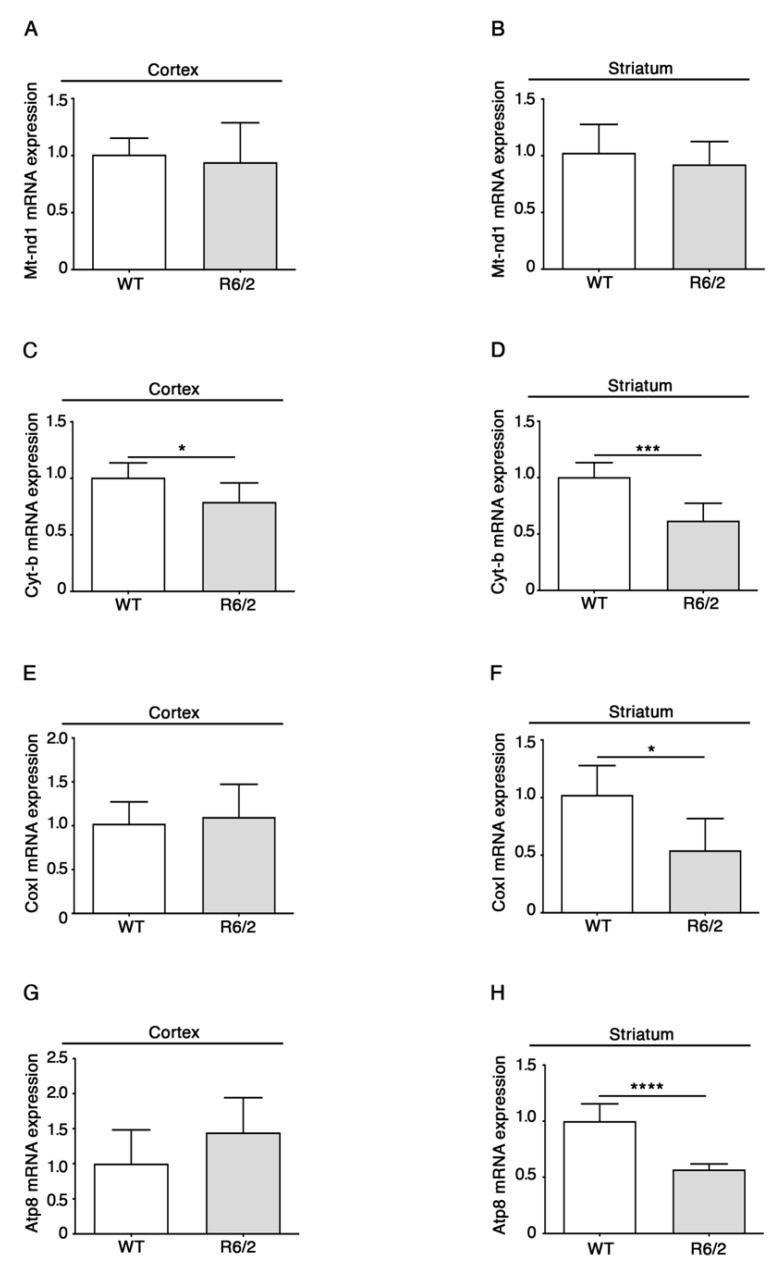
Altered RNA levels of components of the mitochondrial-encoded ETS complex and ATP-synthase genes in brains of 11-week-old R6/2 mice. qPCR analysis of both cortical and striatal Mt-nd1 (**A**,**B**), Cyt-b (**C**,**D**), Cox I (**E**,**F**), Atp8 (**G**,**H**) are presented. *N* = 6 for each group of mice. Data are represented as mean ± SD. * *p* < 0.05; *** *p* < 0.001, **** *p* < 0.0001 (Unpaired *t*-test). The raw data used for generating the figure are reported in the Supplementary Materials Table S2.

qPCR analyses showed no difference between HD and control animals for complex I gene Nd1 and a significant reduction in Cyt-b (complex III) in both the cortex and striatum of R6/2 mice (Figure 2C,D).

Moreover, similar to what was observed for the nuclear-encoded genes, RNAs from the mitochondrially encoded complex IV subunit Cox I (Figure 2E,F), as well as the ATP-synthase subunit Atp8 (complex V) (Figure 2G,H), were significantly decreased only in the striatum of HD mice compared to controls.

These results indicate that RNA levels of ETS complexes and ATP-synthase are differently affected in the cortex and striatum of R6/2 mice (see Table 1). They also suggest that mitochondrial and nuclear transcription are both impaired for some of the investigated genes, but our data do not confirm mitochondria-selective transcription deficits, at least for striatum.

### 3.3. Levels of ETS Proteins Are Differentially Expressed in the Brain of R6/2 Mice

Next, we investigated whether protein levels of components of the mitochondrial ETS were similar in the two vulnerable brain regions (cortex and striatum) of symptomatic R6/2 and WT mice, as the previously described similar resolution respirometry results between the genotypes [28] would suggest.

To determine whether the altered RNA levels in the R6/2 cortex and striatum were associated with changes in the corresponding protein levels, we performed immunoblotting analyses on some of the most interesting gene products identified in our RNA screen from Figure 1.

Based on the important role of complex II in HD (see above), one of the analyzed proteins was SDHA, one of the four proteins (all encoded by nuclear DNA) that constitutes the core complex II. In addition, we analyzed protein levels of COX IV (also expressed from nuclear DNA), which were also specifically reduced in R6/2 striatum. As a supposedly less severely affected ETS component based on the RNA results, the complex I subunit ND1 (encoded by mitochondrial DNA) was selected as well.

**Figure 3:**
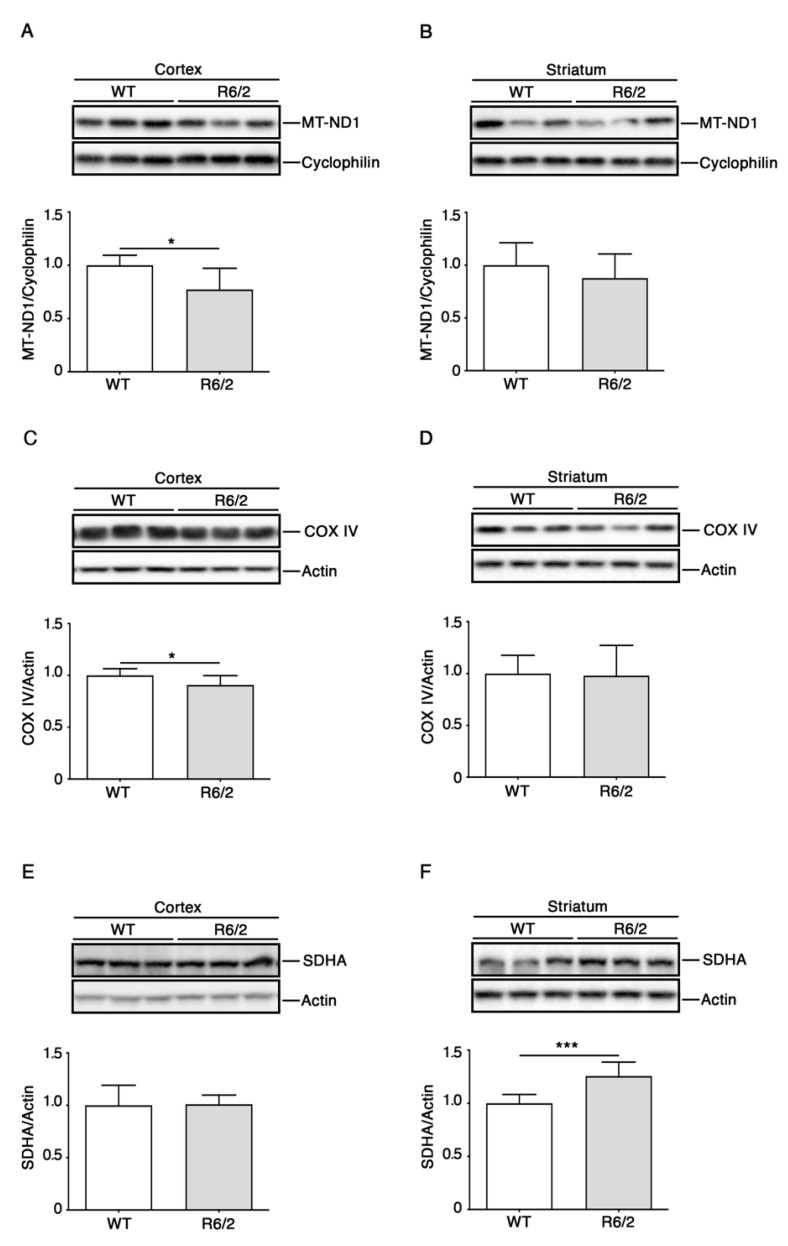
Altered protein levels of components of mitochondrial ETS in R6/2 brain. Representative cropped immunoblotting and densitometric analysis of MT-ND1 (**A**,**B**), COX IV (**C**,**D**) and SDHA (**E**,**F**) in cortical and striatal tissues from 11-week-old R6/2 mice and WT controls. N = 7 for each group of mice. Data are represented as mean ± SD. *, *p* < 0.05; ***, *p* < 0.001 (Unpaired *t*-test). The raw data used for generating the figure are reported in Supplementary Materials Table S3.

Immunoblotting analysis revealed a moderate but significant decrease of ND-1 protein (Figure 3A,B) and COX IV (Figure 3C,D) in the cortex, while SDHA levels were similar in R6/2 as compared to WT mice (Figure 3E,F).

### 3.4. Levels of Cytochrome c Are Reduced in Striatum of R6/2 Mice

An important feature of neurodegenerative diseases is the activation of cell-death signaling under mitochondrial control. Release of cytochrome c from mitochondria is a key step in the apoptotic process, which activates effector caspases and is involved in degeneration of vulnerable neurons in HD models [31,32]. Previously, a shift of cytochrome c from the mitochondrial to the cytoplasmic compartment was reported in HD-patient caudate-putamen and R6/2 striatum at advanced disease stages [32]. Cytochrome c is also a bottleneck component of ETS, shuttling electrons from complex III to complex IV. Here, we also assessed cytochrome c protein levels in the cortex and striatum to determine whether a modulation of the expression of this protein may be involved in HD pathogenesis and potentially affect oxidative phosphorylation or cell death regulation.

**Figure 4:**
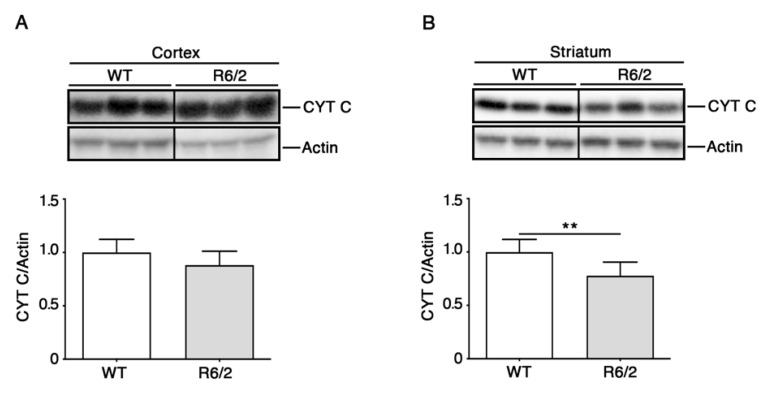
Altered cytochrome c (CYT C) levels in brain tissues of 11-week-old R6/2 mice. Representative cropped immunoblotting and densitometric analysis of cytochrome levels in cortical (**A**) and striatal (**B**) tissues from 11-week-old R6/2 mice and WT controls. In each immunoblotting, all samples were run on the same gel. Non-adjacent samples were separated by a black line. N = 7 for each group of mice. Data are represented as mean ± SD. ** *p* < 0.01 (Unpaired *t*-test). The raw data used for generating the figure are reported in Supplementary Materials Table S4.

As reported in Figure 4, a pronounced decrease of cytochrome c overall protein levels was observed in the striatum, but not in the cortex of R6/2 mice.

## 4. Discussion

Despite much progress in our understanding of the cell-biological underpinnings of HD pathogenesis, the molecular alterations that initiate and facilitate neurodegeneration are still poorly understood. More knowledge of the neurodegenerative processes is, however, integral for the development of effective treatment strategies. The multifaceted mechanisms that constitute HD pathogenesis require detailed investigation of multiple cellular components of different brain regions, cell types and time courses. With cell type specific/single-cell omics technologies, elaborate techniques to assess in vivo functional changes and ever-improving imaging approaches to HD pathogenesis today can be investigated in unprecedented detail. However, these resource-intensive investigations require a basic understanding of the disease processes for an adequate design and reliable animal models. Based on this reasoning, we performed the present explorative study to identify cornerstones for more extensive future research. We employed the R6/2 mouse HD model based on its high validity for human HD [24,33,34]. Based on the reported main proteomic alterations in this model in striatum and cortex [35], we focused on these two brain regions. Our gene products of interest were components of the ETS and ATP-synthase due to well-established deficits in mitochondrial energy metabolism in HD and HD models [36].

The results of the present explorative study suggest brain-region-specific mechanisms to control transcription and translation of mitochondrial ETS components in the HD cortex and striatum. The observation that RNA-expression perturbations occurred not for all genes and both in some mitochondrial and in some nuclear genes in R6/2 striatum is evidence against overall nuclear or mitochondrial transcriptional impairment. While only one mitochondrially encoded RNA was downregulated in the R6/2 cortex, data for more RNAs are needed to determine whether this reflects a specific impairment of mitochondrial transcription. We did not assess functional mitochondrial respiration in the present study. However, our previous study [24] indicated that mitochondrial respiratory capacities in brains of R6/2 model mice are preserved, indicating either compensatory mechanisms or methodological limitations [24].

We speculate that the increased SDHA protein levels in the R6/2 striatum are a compensatory effect for impaired respiratory functionality of complex II (e.g., by mHtt mediated inhibition). The over-abundance of complex II subunit protein levels could induce signals that reduce its transcription in the nucleus.

It has previously been reported that HD models are characterized by a dysregulation of mitochondrial proteins [36]. Some studies observed alterations in ETS components, while others did not. Perluigi et al. [37] reported abnormal changes in protein levels and protein oxidation in 10-week-old R6/2 mouse striatum. Specifically, a decrease of pyruvate dehydrogenase levels as compared to 4-week-old R6/2 mice and an increase of dihydrolipoamide S-succinyltransferase (vs. 4-week-old R6/2 mice) and aspartate aminotransferase (vs. age-matched controls) was observed [37]. These authors, however, did not report significant alterations of components of the ETS. In a 12-week-old R6/2 cortex, an upregulated CYT-b protein (mitochondrially encoded subunit of complex III) was reported, which is in contrast to the reduced RNA levels of that subunit in our study in both the R6/2 cortex and striatum. Similarly, while we observed reduced RNA levels of ATP-synthase subunits in the striatum but not in the cortex, Agrawal and Fox [38] observed reduced protein levels of other ATP-synthase subunits (alpha and delta) in the cortex.

In the study of Damiano et al. [14], the iron-sulfur (but not the FAD-containing catalytic 70 kDa) subunit of complex II was strongly reduced in the striatum of the R6/1 HD model mice at 16 weeks of age. Conversely, an upregulation of complex II activity was reported in both the HD patient and Q175 mouse HD model cortex [39]. In our study, the complex II subunit SDHA was downregulated on the RNA and upregulated on the protein level in the R6/2 striatum without observable differences in the cortex. Complex II (succinate dehydrogenase) has several unique features within the ETS. As mentioned, it is the only ETS complex entirely encoded by nuclear DNA. Furthermore, it is also the only ETS complex that in parallel contributes to the TCA cycle by catalyzing the oxidation of succinate to fumarate and together with the pro-inflammatory potential of succinate may have to be even more tightly regulated than the other components of the mitochondrial respiratory chain [40]. Supporting this notion, aberrant complex II activity is also associated with oxidative stress: reverse electron transport occurs if electrons are transported from coenzyme Q to complex I, for example, by elevated complex II activity. This stimulates the excessive generation of reactive oxygen species [41].

How may the inconsistent findings on the regulation of ETS components be explained? HD pathology may be characterized by a non-linear, dynamic progression of protein expression alterations [42]. Zabel et al. found a marked upregulation of proteins involved in glycolysis and gluconeogenesis that was evidenced already in 2-week-old R6/2 whole brains and was continuously observed until mice were 10 weeks old, after which a partial reversal of this trend occurred [42]. This effect was associated with an early upregulation of key enzymes of the TCA cycle in young R6/2 brains.

This indicates that snapshots—even several—of specific time points (e.g., of RNA, protein or metabolite levels) during HD pathogenesis may be not sufficient to capture the complexity of the disease progression.

Here, we also investigated the possibility of divergent levels of RNAs vs. proteins. Such divergences could be indicative of impaired nuclear or mitochondrial DNA transcription processes or of changes in mitochondrial import. However, RNA and protein levels did not correspond well for the analyzed gene products. In fact, we observed downregulated protein levels of ND1 and COX IV in the R6/2 cortex, whereas the respective RNA levels were not different as compared to wild-type mice. Increased SDHA-protein levels in the R6/2 striatum were even contrasted by reduced RNA levels. The potential regulation of mRNA and protein in the opposite direction during HD pathogenesis has previously been reported [42] and again may be indicative for phasic regulation of transcription and translation during HD progression.

While the present study highlights the differential regulation of various subunits of all ETS complexes and ATP-synthase in a brain-region-specific manner in R6/2 model mice, several limitations associated with the explorative nature of our approach have to be acknowledged and may be important for future research.

Besides the discussed limitation of snapshot approaches, here, we looked at crude tissues from the observed brain regions. This did not allow us to investigate cell type-specific alterations or obtain direct information on cell-compartment effects. The cell type specificity in HD progression is becoming increasingly acknowledged. For example, specific proteomic alteration of astrocytes indicate a prominent role of this cell type in HD [35]. In support of this observation, recently a metabolic shift has been reported for astrocytes in HD striatum from glycolysis to fatty acid oxidation that may be prominently involved in the pathogenesis [43]. These findings highlight the importance of the contribution of different cell types.

Although a great focus in HD research has been on pathological gain-of-function effects of mutated Htt, it will be important to not neglect the possibility of loss-of-function effects due to the mutation and/or aggregation of the protein. This loss-of-function may also concern an important role of the wild-type Htt protein in energy metabolism, as recently indicated by research in mouse cardiocytes [23].

The possibility of impaired mito-nuclear signaling that may contribute to the regulation of SDHA in the striatum again supports the potential wave-like patterns [42] of transcriptional (and translational) control. Organelle-specific impairments and potential deficits in inter-organelle communication should be taken into account in future studies.

Taken together, our results in light of the previous literature have important implications for future investigations. First, single-cell or cell type-specific omics approaches will help us to obtain a more complete overview [44] on molecular changes during HD pathogenesis. It will be of great importance to consider the specific temporal patterns of molecular alterations for these studies; snapshots of individual time points may not accurately capture the disease progression.

Second, the possibility of fluctuating, wave-like patterns of molecular alterations highlight the importance of using in vivo technologies to better understand the temporal evolution of HD pathogenesis. For example, in vivo electrophysiology or imaging approaches will provide clearer rationales for when the relevant omics-studies should be performed.

In this study, we observed analyzed tissues from both male and female R6/2 mice and controls. Although we did not notice marked sex-dependent differences, future studies specifically designed to investigate potential differences in HD pathology due to sex will be of great importance.

## 5. Conclusions

In conclusion, the present approach allowed us to identify potential mechanisms that will enable the appropriate design for much-needed single-cell or cell type-specific omics analysis that—in combination with in vivo assessment of mitochondrial functionality and the analysis of organelle-specific alterations—will inform extensively on HD-pathology–associated mitochondrial alterations.

## Figures and Tables

**Table 1:** Summary of altered RNA levels of components of mitochondrial electron transport system and ATP-synthase in R6/2 brains. Red shading indicates reduced RNA levels in R6/2 cortex or striatum as compared to wild-type; grey shading indicates no differences.

		Nuclear RNA			Mitochondrial RNA	
ETS Complex	Subunit	Cortex	Striatum	Subunit	Cortex	Striatum
CI	Ndufa2			Nd1		
CII	Sdha				--	--
CIII	Uqcrc			Cyt-b		
CIV	Cox-IV			Cox-I		
CV	Atp5b			Atp8		

## Data Availability

The research data used for generating the figures are available as Supplementary Materials.

## Supplementary Materials

The following are available online at https://www.mdpi.com/article/10.3390/brainsci11101267/s1, Table S1: Research raw data used for generating Figure 1. Table S2: Research raw data used for generating Figure 2. Table S3: Research raw data used for generating Figure 3. Table S4: Research raw data used for generating Figure 4.
