# Supplementary material for: Brain Region and Cell Compartment Dependent Regulation of Electron Transport System Components in Huntington’s Disease Model Mice
Source: Brain Sci. 2021 Sep 24;11(10):1267. doi: 10.3390/brainsci11101267 (PMC8533690; doi:10.3390/brainsci11101267)
Supplement: Supplementary file 1 [file brainsci-11-01267-s001.zip › brainsci-1383121-supplementary.pdf]

Supplementary Materials

Brain region and cell compartment dependent regulation of electron transport system components in Huntington's disease model

Johannes Burtscher [1, 2, *], Giuseppe Pepe [3], Federico Marracino [3], Luca Capocci [3], Susy Giova [3], Grégoire P. Millet [1,2], Alba Di Pardo [3] and Vittorio Maglione [3, *]

1 Institute of Sport Sciences, University of Lausanne, CH-1015, Lausanne, Switzerland
2 Department of Biomedical Sciences, University of Lausanne, CH-1015, Lausanne, Switzerland; gregoire.millet@unil.ch
3 IRCCS Neuromed, 86077, Pozzilli (IS), Italy; g.pepe1604@gmail.com; federicomarracino@gmail.com; luca.capocci@virgilio.it; susygiova95@gmail.com; dipardoa@hotmail.com
* Correspondence: J.B.; Johannes.burtscher@unil.ch; Tel. +41 21 692 37 97. V.M.; vittorio.maglione@neuromed.it; Tel. +39 0865 915212

Table S1. Research raw data used for generating Figure 1

CORTEX				STRIATUM		
Fig. 1 A				Fig. 1B		
Ndufa2 mRNA expression	WT	R6/2		Ndufa2 mRNA expression	WT	R6/2
	0,999323	1,03892			1,022098	0,920845
	1,000677	1,064132			0,966433	0,975128
	1,002	1,048			1,04056	0,938552
	0,977	1,182			0,97091	0,921334
	0,828	1,837			0,915	1,018
	1,234	1,071			1,093	1,429
Fig. 1 C				Fig. 1D		
Sdha mRNA expression	WT	R6/2		Sdha mRNA expression	WT	R6/2
	0,957772	1,095153			0,955269	0,575064
	1,042228	1,120995			0,98652	0,589745
	0,925	0,858			0,975857	0,558891
	1,433	1,149			1,082354	0,569422
	0,782	0,723			0,947	0,807
	0,964	1,278			1,055	0,664
Fig. 1E				Fig. 1F		
Uqcrc mRNA expression	WT	R6/2		Uqcrc mRNA expression	WT	R6/2
	1,011743	0,883678			0,973225	0,728546
	0,988257	0,946525			1,008133	0,774207
	1,051	0,806			1,011504	0,731376
	1,413	0,627			1,007138	0,747333
	0,613	1,179			0,93	1,127
	1,098	0,813			1,075	0,991
Fig. 1G				Fig. 1H		
CoxIV mRNA expression	WT	R6/2		CoxIV mRNA expression	WT	R6/2
	0,973	0,879			0,805	0,562
	0,727	0,983			0,986	0,072
	1,024	1,309			0,982	0,415
	1,157	2,075			1,293	0,855
	1,193	0,841			0,8	0,627
					1,241	0,459
Fig. 1I				Fig. 1L		
Atp5b mRNA expression	WT	R6/2		Atp5b mRNA expression	WT	R6/2
	1,015924	0,742908			0,998257	0,221411
	0,984076	0,781149			1,01089	0,246417
	1,356	0,857			0,990206	0,228333
	0,834	1,028			1,000646	0,373377
	0,867	1,177			0,884	0,837
	1,02	0,993			1,131	1,015

Table S2. Research raw data used for generating Figure 2

CORTEX				STRIATUM		
**Fig. 2A**				**Fig. 2B**		
**Mt-nd1 mRNA expression**	**WT**	**R6/2**		**Mt-nd1 mRNA expression**	**WT**	**R6/2**
	0,979635	0,800353			1,26	0,874
	1,020365	0,896718			0,916	0,94
	1,183	1,397			0,646	1,306
	0,78	1,057			1,341	0,897
	1,138	0,38			0,996	0,802
	0,953	1,129			1,004	0,726
**Fig. 2C**				**Fig. 2D**		
**Cyt-b mRNA expression**	**WT**	**R6/2**		**Cyt-b mRNA expression**	**WT**	**R6/2**
	1,003767	0,675736			1,054561	0,70678
	0,996233	0,782542			0,932838	0,750766
	1,069	0,92			1,01964	0,693841
	0,966	1,031			0,992962	0,720999
	0,806	0,779			1,208	0,44
	1,203	0,563			0,828	0,407
**Fig. 2E**				**Fig. 2F**		
**CoxI mRNA expression**	**WT**	**R6/2**		**CoxI mRNA expression**	**WT**	**R6/2**
	1,252	1,526			0,851	0,466
	1,046	1,103			1,175	0,336
	0,787	0,979			0,8	0,136
	0,767	0,845			0,821	0,723
	0,918	0,615			1,446	0,835
	1,376	1,539			1,052	0,762
**Fig. 2G**				**Fig. 2H**		
**Atp8 mRNA expression**	**WT**	**R6/2**		**Atp8 mRNA expression**	**WT**	**R6/2**
	1,59696	1,853044			1,220905	0,525572
	0,894092	1,83867			1,09382	0,553616
	1,118539	1,072715			0,78726	0,538768
	0,342199	0,858035			0,898015	0,538509
	0,606679	1,078178			1,052	0,622
	1,441531	1,981206			0,951	0,639

Table S3. Research raw data used for generating Figure 3

CORTEX				STRIATUM		
Fig. 3A				Fig. 3B		
MT-ND1/Cyclophilin	WT	R6/2		MT-ND1/Cyclophilin	WT	R6/2
	0,961765	0,836553			1,09576	0,610547
	1,037712	0,574269			1,115783	0,596578
	1,000523	0,636901			0,788457	1,034635
	1,190114	0,516035			0,962869	0,781051
	0,907976	1,010009			1,355555	1,137131
	0,926619	0,988174			0,722447	0,858158
	0,975292	0,848936			0,959129	1,127828
Fig. 3C				Fig. 3D		
COX IV/Actin	WT	R6/2		COX IV/Actin	WT	R6/2
	0,903239	0,891756			0,910788	0,837339
	1,08954	0,927489			1,212434	0,564927
	1,007221	1,018338			0,876778	0,972696
	1,066199	0,950507			1,200473	0,819992
	0,955302	0,975597			1,130165	1,482757
	1,010645	0,845371			0,770803	0,996034
	0,967854	0,751749			0,898559	1,189181
Fig. 3E				Fig. 3F		
SDHA/Actin	WT	R6/2		SDHA/Actin	WT	R6/2
	1,370232	0,891832			0,970698	1,233645
	0,790465	1,023235			1,075776	1,103651
	0,839303	1,155265			0,953527	1,144806
	0,962355	0,950075			0,918567	1,256223
	0,984246	1,080941			1,048364	1,51135
	1,098202	0,964217			0,909206	1,290444
	0,955197	1,011324			1,123864	1,259883

Table S4. Research raw data used for generating Figure 4

CORTEX				STRIATUM		
Fig. 4A				Fig. 4B		
CYT C/Actin	WT	R6/2		CYT C/Actin	WT	R6/2
	1,121222	0,738428			1,056743	0,972079
	0,973639	0,808403			1,104674	0,677461
	0,905139	0,750076			0,838583	0,873621
	1,14713	0,834764			1,054995	0,703214
	0,858297	1,066651			1,141294	0,883377
	1,107892	0,988613			0,886099	0,67839
	0,886681	0,983258			0,917611	0,65934
